# The copper-associated protein STEAP2 correlated with glioma prognosis and immune infiltration

**DOI:** 10.3389/fncel.2022.944682

**Published:** 2022-08-18

**Authors:** Xu Wang, Mingzhi Han, Songyu Chen, Yanfei Sun, Ruirong Tan, Bin Huang

**Affiliations:** ^1^Department of Neurosurgery, Qilu Hospital, Cheeloo College of Medicine and Institute of Brain and Brain-Inspired Science, Shandong University, Jinan, China; ^2^Jinan Microecological Biomedicine Shandong Laboratory and Shandong Key Laboratory of Brain Function Remodeling, Jinan, China; ^3^Medical Integration and Practice Center, Cheeloo College of Medicine, Shandong University, Jinan, China; ^4^Department of Neurosurgery, Shanghai Tenth People’s Hospital, Tongji University School of Medicine, Shanghai, China; ^5^Translational Chinese Medicine Key Laboratory of Sichuan Province, Sichuan Institute for Translational Chinese Medicine, Sichuan Academy of Chinese Medicine Sciences, Chengdu, China

**Keywords:** glioma, copper associated protein, STEAP2, immune infiltration, tumor mutation burden (TMB)

## Abstract

High-grade glioma is characterized by cell heterogeneity, gene mutations, and poor prognosis. Abnormal copper homeostasis affects the pathogenesis of glioma, but the underlying mechanisms and involved proteins are unknown. Here, we selected 90 copper-related proteins and verified their expression differences in glioma and normal tissues in the TCGA cohort followed by GO and KEGG clustering analyses. We then developed and validated a prognostic model. Moreover, we examined the mutation burden of copper-related proteins and discussed the differences in the immune microenvironment in the high- and low-risk groups. Furthermore, we focused on STEAP2 and demonstrated that STEAP2 expression was relatively low in tumor tissues compared to normal tissues, implying a favorable prognosis. Our findings provide a foundation for future research targeting copper-related proteins and their immune microenvironment to improve prognosis and responses to immunotherapy.

## Introduction

Glioblastoma multiforme (GBM) is the most malignant high-grade glioma with a survival time of 12–18 months (Louis et al., 2021). Classical therapies for GBM show limited effects on the improvement of overall survival (OS) (Wu et al., 2021). Recent studies have shown that high-grade glioma is characterized by cell heterogeneity, gene mutations, and poor prognosis (Zhang et al., 2021). Thus, new therapies based on a combination of genetics and immunology have been developed, providing more options (Kitamura et al., 2010; Qiao et al., 2016; Huang et al., 2020; Qi et al., 2020; Banerjee et al., 2021). Through the study of molecular and genomic features, the tumor microenvironment and tumor-host interactions have become important for the development of new potential therapies.

In physiological conditions, copper was always bound to proteins to prevent uncontrolled redox activity. Once transported into the cytoplasm by Ctr1, copper was involved in the control of oxidative stress through CCS and SOD1, and also implicated in mitochondrial respiration. Copper functioned on different molecular pathways leading to a proangiogenic response necessary for carcinogenesis processes and copper accumulation could be observed in cancer cells. However, the relationship between copper levels and cancer remains still unexplained up to now (Lelievre et al., 2020). Abnormal copper homeostasis affects the pathogenesis of tumors. Copper is involved in cancer development and progression, and it facilitates cancer growth, angiogenesis, and metastasis, leading to the development of copper coordination compounds for anticancer therapies (Babak and Ahn, 2021; Shanbhag et al., 2021). Additionally, copper disturbs normal metabolic processes and induces cuproptosis, showing a multifaceted role (Lelievre et al., 2020).

Copper-related proteins are crucial for the performance of cellular copper, such as copper transportation, binding, and cuproptosis (Shanbhag et al., 2019; Tsvetkov et al., 2022). The effects of copper-related proteins on tumor progression have been revealed in many cancers, such as breast cancer, colorectal cancer, and melanoma (Blockhuys and Wittung-Stafshede, 2017; Lelievre et al., 2020). Although copper-related biomarkers were not directly applicable to clinical applications yet, there have been a number of promising research. ATOX1 expression levels were indicated as a potential prognostic biomarker for ER-positive subtypes of breast cancer (Blockhuys et al., 2020). ATP7A expression may also be a predictive biomarker of drug resistance and a negative prognostic factor for survival in ovarian cancer patients treated with platinum-based chemotherapy (Samimi et al., 2004). Recent studies have demonstrated that the majority of copper-related proteins are pro-oncogenic factors, while the function of certain proteins depends on their context. For example, SPARC is associated with highly aggressive phenotypes in glioma (Schultz et al., 2012), but it functions as a tumor suppressor in medulloblastoma (Bhoopathi et al., 2011). Thus, further studies are required to gain deeper insight into the function of copper-related proteins. Elucidating the functions and detailed mechanisms of these key proteins is important for understanding the performance of copper and providing new strategies for copper-based cancer therapies. However, the underlying mechanisms and involved proteins are unknown, especially in glioma.

Here, we selected 90 copper-related proteins and verified their expression differences in variable grades of glioma, and compared them to normal tissues. We identified the molecular characteristics and prognostic values of these differentially expressed copper-related proteins, and we developed a prognostic model and validated it using the Rembrandt database. Moreover, we identified the tumor mutational burden of copper-related proteins and discussed the differences in the immune microenvironment in the high-risk and low-risk groups. Furthermore, we focused on six-transmembrane epithelial antigen of prostate 2 (STEAP2), a prognosis-related protein, among 13 differentially expressed copper-related proteins, and we demonstrated that STEAP2 had relatively low expression in tumor tissues compared to normal tissues, implying a favorable prognosis. Our findings provide a foundation for future research targeting copper-related proteins and their immune microenvironment to improve prognosis and responses to immunotherapy.

## Materials and methods

### TCGA and rembrandt glioma cohorts

The protein expression data of glioma patients were obtained from TCGA^1^ and REMBRANDT^2^ databases. The tumor mutational burden (TMB) and mutation counts were computed from somatic mutation frequencies. The related GO functional enrichment and KEGG pathway enrichment analyses were performed using the GO database^3^ and the KEGG database^4^, respectively.

### Construction of the prognostic model

Hazard ratios were calculated using a Cox proportional hazard model. Based on the expression data of 13 prognosis-related genes, the risk score of TCGA glioma patients was obtained, and the patients were divided into low- and high-risk groups according to the median score. The prognostic model was further validated using the Rembrandt cohort.

### Immune infiltration estimations

To estimate immune infiltration, we used CIBERSORT^5^ to compare immune cell-related genes between samples. We used the chi-square test to identify the difference in the tumor immune microenvironment between the high- and low-IRGPI risk groups.

### Gene expression analysis

We inputted STEAP2 into the “Gene_DE” module of the TIMER2 (Tumor Immune Estimation Resource, version 2) webserver^6^ and evaluated the expression difference in STEAP2 between tumor and adjacent normal tissues for the various tumors or specific tumor subtypes in TCGA database. We utilized the UALCAN portal^7^, an interactive web resource for analyzing cancer omics data, to conduct protein expression analysis of the Clinical proteomic tumor analysis consortium (CPTAC) dataset (Chen et al., 2019).

### Survival prognosis analysis

We used the “Survival Map” module of GEPIA2 (Tang et al., 2019) to obtain the OS and disease-free survival (DFS) significance map data of STEAP2 across all TCGA tumors. High (50%) and low (50%) cutoff values were used as the expression thresholds for dividing the high-expression and low-expression cohorts. The log-rank test was used in the hypothesis test, and the survival plots were obtained through the “Survival Analysis” module of GEPIA2.

### Genetic alteration analysis

We utilized the cBioPortal webserver^8^ (Cerami et al., 2012; Gao et al., 2013) to query the genetic alteration characteristics of STEAP2 in “TCGA Pan Cancer Atlas Studies.” The results of the alteration frequency, mutation type, and copy number alteration (CNA) across all TCGA were observed in the “Cancer Types Summary” module. The mutated site information of STEAP2 was displayed in the schematic diagram of the protein structure or the three-dimensional (3D) structure via SWISS-MODEL.^9^ The sequence of the amino acid residues was obtained from UniProt.^10^

## Results

### Summary of copper-related proteins

**FIGURE 1:**
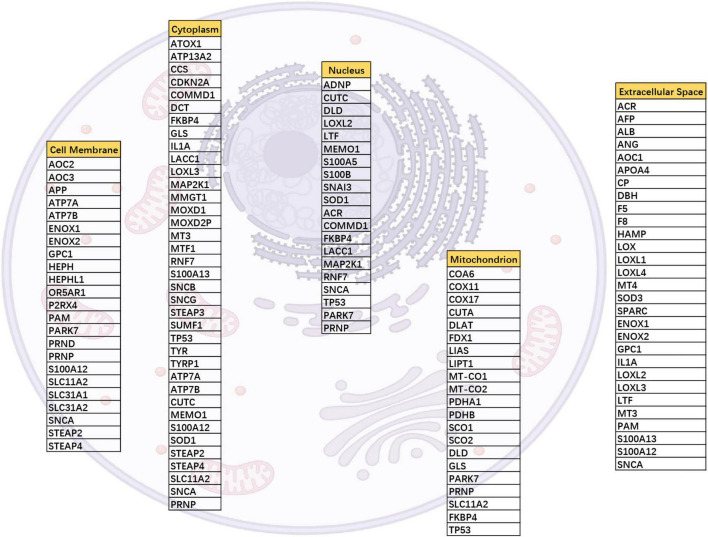
Cellular localization of 90 copper-associated proteins. The distribution of copper-associated proteins was obtained from UniProt, and one protein may localize in various locations. For example, SNCA mapped to the cell membrane, cytoplasm, nucleus, and extracellular space.

To search for copper-related proteins involved in glioma progression, we first identified 90 copper-related proteins associated with transportation into cells and the function of copper ions. We selected 61 copper ion-binding proteins and 16 copper ion transport proteins based on Gene Ontology analysis as well as previously confirmed copper-related proteins, including pyroptosis-related proteins (Blockhuys et al., 2017), which resulted in a list of 90 copper-related proteins. We searched the subcellular locations of these proteins through GeneCards and UniProt (Figure 1). We next investigated the roles that these proteins play in glioma progression.

### Prognostic and molecular features of differentially expressed copper-related proteins

**FIGURE 2:**
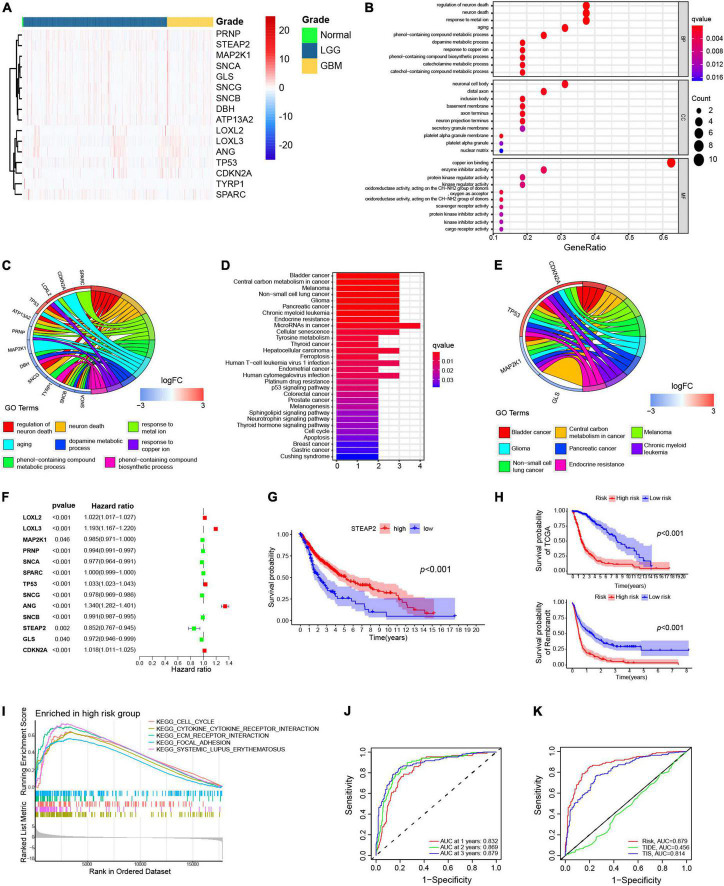
Differentially expressed copper-related genes in GBM. **(A)** Heatmap displaying all differentially expressed copper-related genes among 169 GBM samples (orange), 529 LGG samples (blue), and 5 normal tissue samples (green). **(B,C)** Gene Ontology (GO) enrichment analysis of the differentially expressed copper-related genes (*p* < 0.05). **(D,E)** Kyoto Encyclopedia of Genes and Genomes (KEGG) pathway analysis of differentially expressed copper-related genes (*p* < 0.05). **(E)** Univariate Cox analysis of 13 prognosis-related genes. **(F)** K–M survival analysis of TCGA samples based on STEAP2 expression (*p* < 0.05). **(G)** K–M survival analysis of the high-risk and low-risk subgroups in TCGA cohort based on the prognostic model. **(H)** K–M survival analysis of the high-risk and low-risk subgroups in the Rembrant cohort (*n* = 356) based on the prognostic model. **(I)** Gene sets enriched in the high-risk subgroup (*p* < 0.05 and FDR < 0.25). **(J)** ROC curve for 1-, 2-, and 3-year survival of the prognostic model in TCGA cohort. **(K)** Comparison of different prognostic models.

To select the crucial copper-related proteins involved in the malignant progression of glioma, we obtained the expression values of 90 genes in LGG and GBM samples from the TCGA cohort. The differential expression analysis (698 glioma vs. 5 normal samples) indicated a total of 16 differentially expressed genes, including 6 upregulated genes and 10 downregulated genes in the tumor samples compared to the normal samples (Figure 2A). Functional enrichment analysis showed that the differentially expressed genes were significantly associated with 8 GO terms and 8 KEGG pathways (Figures 2B–E).

To determine the independent prognostic genes, a univariate Cox regression analysis for OS was performed among the differentially expressed copper-related genes. As shown in Figure 2F, 13 of the 16 genes were independently associated with prognosis, and only three genes (LOXL3, ANG, and STEAP2) significantly affected the OS of patients with glioma. Moreover, only high expression of STEAP2 increased the OS of GBM patients (Figure 2G). We next constructed a prognostic model based on the Cox proportional hazard model of the 13 genes and divided all samples into two groups according to risk score with the median value as the cutoff value. We then verified the survival outcomes of the two groups using TCGA and Rembrandt (356 samples) cohorts. The results indicated that high-risk patients had a worse OS than low-risk patients (Figure 2H).

GSEA was performed to determine the enriched gene sets in the risk score subgroups. The high-risk group gene set was enriched in the cell cycle, cytokine–cytokine receptor interaction, ECM receptor interaction, focal adhesion, and systemic lupus erythematosus (Figure 2I). Additionally, the prognostic model was verified by ROC curve analysis. The AUC values for 1-, 2-, and 3-year survival were 0.832, 0.869, and 0.879, respectively (Figure 2J), indicating that the prognostic model predicts the survival of patients. We also compared the predictive ability of different models. The AUC values of the copper model risk, TIDE, and TIS curves were 0.879, 0.456, and 0.814, respectively, which indicated that our prediction model was better than TIDE and TIS for glioma (Figure 2K). Together, these findings confirmed the prognostic values of copper-related proteins.

### Tumor mutational burden and immune characteristics of differentially expressed copper-related proteins

**FIGURE 3:**
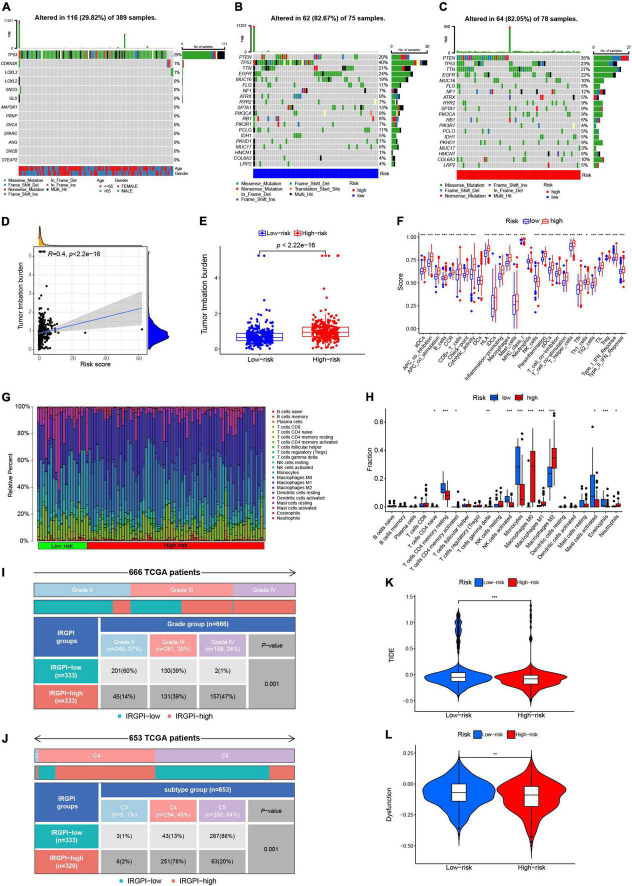
Landscape of the TME and the immune infiltration of different subgroups. **(A)** Tumor mutation burden of copper-related proteins. Mutated genes (rows, top 13) are ordered by mutation rate, and samples (columns) are arranged to emphasize mutual exclusivity among mutations. The right shows the mutation percentage, and the top shows the overall number of mutations. The color coding indicates the mutation type. **(B,C)** Significantly mutated genes in the mutated GBM samples of different subgroups. Mutated genes (rows, top 20) are ordered by mutation rate. **(D)** Correlation analysis between risk score and total mutational burden. **(E)** Total mutational burden of the high-risk and low-risk groups. **(F)** Differential analysis of immune-related biological processes in the high- and low-risk groups. **(G,H)** The proportions of TME cells in the high- and low-risk groups in TCGA cohort. **(I,J)** The IRGPI group based on different grades and immune subtypes in the high- and low-risk groups. **(K,L)** TIDE and dysfunction scores in the high-risk and low-risk groups. The scores between the two groups were compared using the Wilcoxon test (ns, not significant; **p < 0.01; ***p < 0.001).

We next analyzed gene mutations to gain further biological insight into the immunological nature of the subgroups. We calculated the TMB of the 13 prognosis-related proteins based on the immune data from the TCGA cohort (Figure 3A). TP53 had the highest mutation percentage (29%), and the main mutation type of TP53 was a missense mutation. We then analyzed gene mutations to gain further biological insight into the immunological nature of the high-risk and low-risk groups. We found that missense variations were the most common mutation type followed by nonsense and frameshift deletions. We then identified the top 20 genes with the highest mutation rates in the high-risk and low-risk groups (Figures 3B,C). Mutations in the TP53 and TTN genes were more common in the high-risk group, while mutations in the PTEN and TTN genes were more common in the low-risk subgroup.

The risk score showed a positive correlation with TMB (Figure 3D). Additionally, the high-risk group showed a high TMB compared to the low-risk group (Figure 3E). The immune function analysis in the high-risk and low-risk groups showed that most immune function processes were more activated in the high-risk group, while only the function of NK cells was more active in the low-risk group (Figure 3F). To analyze the composition and immune cells in the high-risk and low-risk groups, we used the Wilcoxon test to compare the distribution of immune cells in the different groups. We found that M0 macrophages, M1 macrophages and M2 macrophages (*p* < 0.001), gamma delta T cells (*p* < 0.01), activated memory CD4 T cells, and neutrophils (*p* < 0.05) were more abundant in the high-risk group, while resting memory CD4 T cells, activated NK cells, and monocytes were more abundant in the low-risk group (Figures 3G,H).

The proportion of Grade III samples was equally distributed between the two groups; however, there were more Grade IV samples and fewer Grade II samples in the IRGPI-high group than in the IRGPI-low subgroup (*P* < 0.001, X2 test; Figure 3I). Regarding different immune subtypes, there were more C5 subtypes in the IRGPI-low subgroup and more C4 subtypes in the IRGPI-high subgroup (*P* < 0.001, X2 test; Figure 3J).

TIDE was then utilized to examine the potential clinical efficacy of immunotherapy in different risk groupings. Patients with a higher TIDE prediction score had a higher risk of immune evasion, which implied that they were less likely to benefit from ICI therapy. The high-risk cohort had a lower TIDE score than the low-risk subgroup, which indicated that patients in the high-risk category were likely to benefit more from ICI therapy than patients in the low-risk subgroup (Figure 3K). Furthermore, a greater TIDE prediction score was linked to a negative result. Thus, high-risk subgroups with low TIDE scores may have a better prognosis than low-risk subgroups with high TIDE scores. In addition, the low-risk group had a higher score for T-cell malfunction (Figure 3L).

### Prognostic values of STEAP2 in variable grades of glioma

**FIGURE 4:**
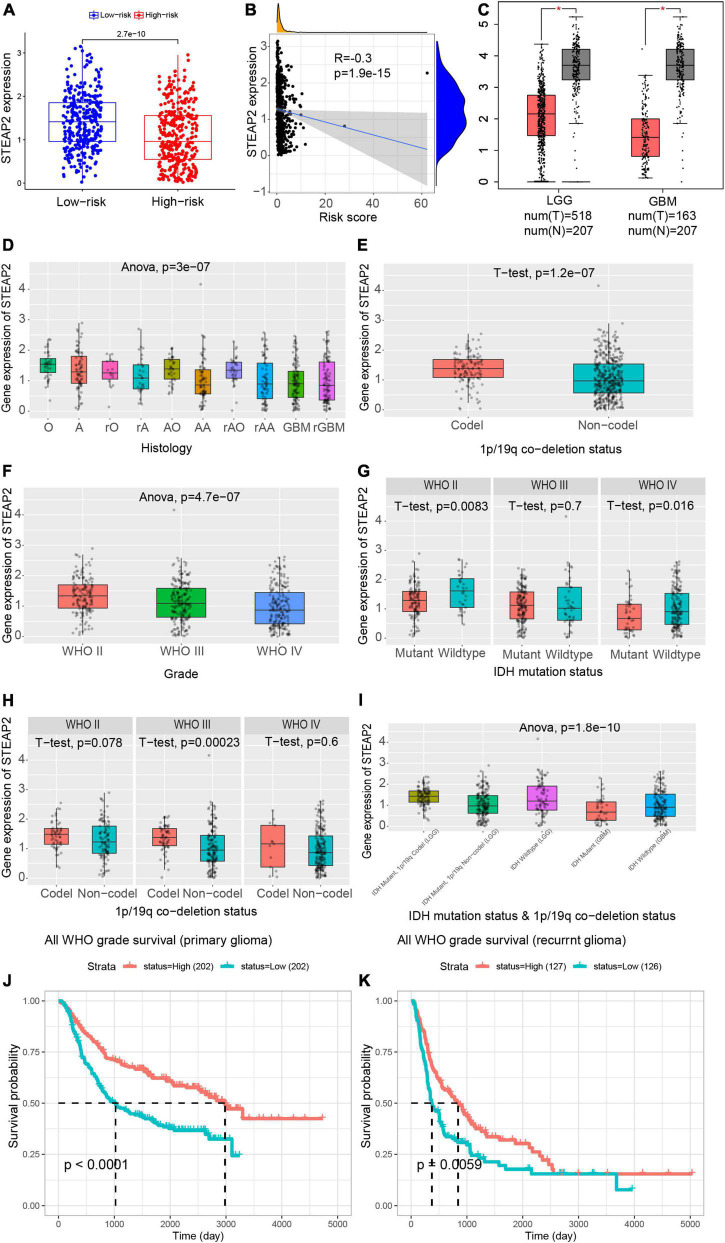
Prognostic values of STEAP2 in variable grades of glioma. **(A)** Expression level of STEAP2 in the high-risk and low-risk groups. **(B)** Correlation analysis between the risk score and STEAP2 level. **(C)** Expression level of STEAP2 in normal and tumor tissues of LGG and GBM patients. **(D)** Expression level of STEAP2 in glioma with different histologies. **(E)** Expression level of STEAP2 in the 1p/19q codeletion and non-codeletion groups. **(F)** Expression level of STEAP2 in different WHO grades of glioma. **(G)** Expression level of STEAP2 in the IDH mutation and wild-type groups of different WHO grades of glioma. **(H)** Expression level of STEAP2 in the 1p/19q codeletion and non-codeletion groups of different WHO grades of glioma. **(I)** Expression level of STEAP2 in the different 1p/19q codeletion and IDH mutation groups of LGG and GBM. **(J)** Relationship between STEAP2 expression levels and overall survival in primary glioma according to the CGGA database. **(K)** Relationship between STEAP2 expression levels and overall survival in recurrent glioma according to the CGGA database.

As mentioned above, high expression of STEAP2 prolonged the OS of glioma patients. The STEAP family has been confirmed to participate in absorbing and reducing iron and copper as well as regulating the growth and proliferation of cancer cells (Sun et al., 2019). STEAP2 has been reported to inhibit epithelial-to-mesenchymal transition of breast cancer and invasion of papillary thyroid cancer (THCA) (Yang et al., 2020; Zhu et al., 2022). The expression level of STEAP2 was lower in the high-risk group than in the low-risk group (Figure 4A), and the risk factor was negatively correlated with the expression of STEAP2 (Figure 4B). To further confirm the function of STEAP2 in glioma, we evaluated the correlation of STEAP2 with the clinicopathological parameters of different grades of glioma using GEPIA. Compared to normal brain tissues, STEAP2 was expressed at lower levels in both LGG and GBM (Figure 4C). As shown in Figure 4D, GBM, recurrent GBM, anaplastic astrocytoma, recurrent anaplastic astrocytoma, and recurrent astrocytoma had lower STEAP2 expression, while oligodendroglioma, astrocytoma, recurrent oligodendroglioma, anaplastic oligodendroglioma, and recurrent anaplastic oligodendroglioma had higher STEAP2 expression. The 1p/19q codeletion is a crucial marker for the measurement of glioma malignancy, and the 1p/19q codeletion group had a higher STEAP2 level (Figure 4E). We then divided the samples into three groups according to WHO glioma grading and found that STEAP2 expression levels decreased with increasing glioma grade (Figure 4F). Moreover, we found that STEAP2 had lower expression in the IDH mutant group and the non1p/19q codeletion group (Figures 4G,H). Considering multiple factors, the STEAP2 level was lower in the IDH mutant and 1p/19q codeletion group in LGG compared to the other two groups, while the expression of STEAP2 was lower in the IDH mutant group of GBM (Figure 4I). All WHO grade survival analyses by CGGA^11^ confirmed that a high level of STEAP2 predicted a better prognosis and longer overall survival in both primary and recurrent tumors (Figures 4J,K). Thus, these findings suggested that STEAP2 indicates a favorable prognosis in glioma and that it may be an antitumor factor.

### Expression level of STEAP2 in different tumors and correlation with survival

**FIGURE 5:**
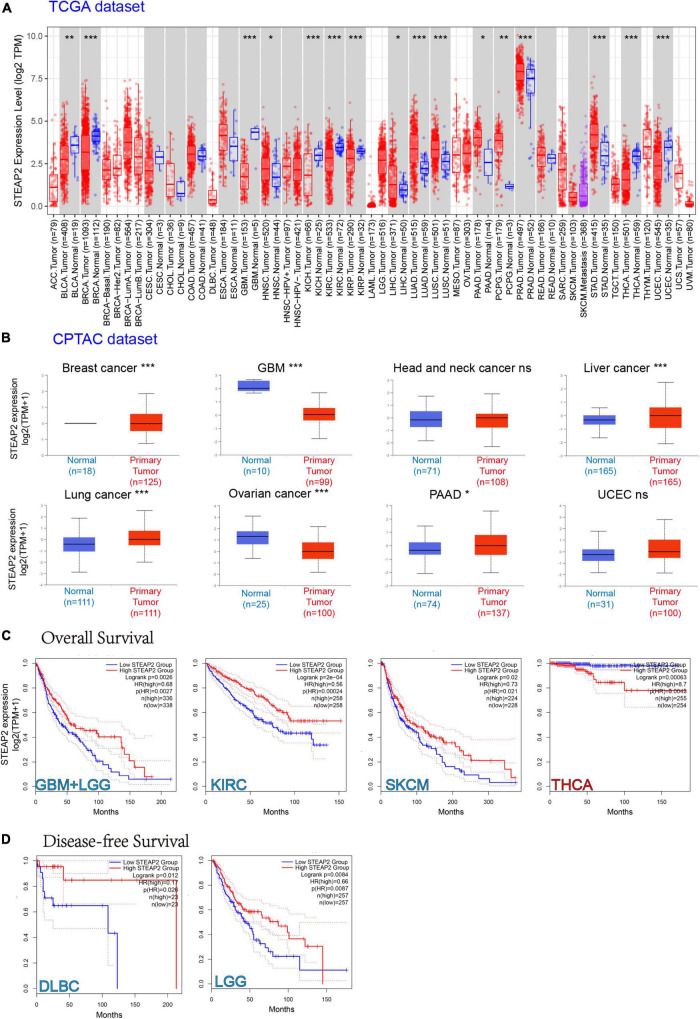
Expression level of STEAP2 in different tumors and survival prognosis. **(A)** The expression status of the STEAP2 gene in different cancers or specific cancer subtypes was analyzed through TIMER2. *P < 0.05; **P < 0.01; ***P < 0.001. **(B)** Expression level of STEAP2 total protein in normal tissue and primary tissue of breast cancer, liver cancer, lung cancer, PAAD, UCEC, GBM, and ovarian cancer according to the CPTAC dataset. *P < 0.05; ***P < 0.001. **(C,D)** Overall survival and disease-free survival analyses of different tumors based on STEAP2 gene expression in TCGA. The survival map and Kaplan–Meier curves show positive results.

To understand the role of human STEAP2 in cancer progression, we focused on the functions of STEAP2 across cancers. To analyze STEAP2 expression in various cells and non-tumor tissues, we used TIMER2 for comparison across TCGA cancer types. As shown in Figure 5A, the expression levels of STEAP2 in the tumor tissues of breast invasive carcinoma (BRCA), GBM, kidney chromophobe (KICH), kidney renal clear cell carcinoma (KIRC), kidney renal papillary cell carcinoma (KIRP), thyroid cancer (THCA), uterine corpus endometrial carcinoma (UCEC) (*p* < 0.001), and bladder cancer (BLCA) (*p* < 0.01) were lower than those in the corresponding control tissues. However, the expression levels of STEAP2 in the tumor tissues of lung adenocarcinoma (LUAD), lung squamous cell carcinoma (LUSC), prostate cancer (PRAD), stomach cancer (STAD) (*p* < 0.001), pheochromocytoma and paraganglioma (PCPG) (*p* < 0.01), head and neck squamous cell carcinoma (HNSC), liver hepatocellular carcinoma (LIHC), and pancreatic adenocarcinoma (PAAD) (*p* < 0.05) were higher than those in the corresponding control tissues.

The CPTAC dataset revealed that STEAP2 total protein expression was higher in primary tissues of liver cancer, lung cancer (*p* < 0.001), and PAAD (*p* < 0.05) compared to normal tissues, but it was lower in GBM (*p* < 0.001) and ovarian cancer (Figure 5B, *p* < 0.05) compared to normal controls.

We separated the cases into high-expression and low-expression groups based on STEAP2 expression levels and evaluated the relationship between STEAP2 expression and prognosis in patients with various malignancies, primarily utilizing TCGA and GEO databases. As shown in Figure 5C, high STEAP2 expression was associated with poor OS for cancers of THCA (*p* = 0.00063) but with favorable progression for GBM + LGG (*p* = 0.0026), KIRC (*p* = 0.0002), and SKCM (*p* = 0.02). Moreover, we showed a correlation between high STEAP2 expression and a positive prognosis of DFS for TCGA cases of DLBC (*p* = 0.012) and LGG (*p* = 0.0084) (Figure 5D). STEAP2 has been reported to have a reverse function in different cancers, such as a cancer-promoting effect in PRAD (Burnell et al., 2018) and a tumor-suppressive function in breast cancer (Yang et al., 2020). While the dual role of certain proteins in different cancers is not unusual (Reina-Campos et al., 2019), it depends on their structure (Gomes et al., 2012) and cellular context.

### Mutation feature of STEAP2 in different tumors of TCGA

**FIGURE 6:**
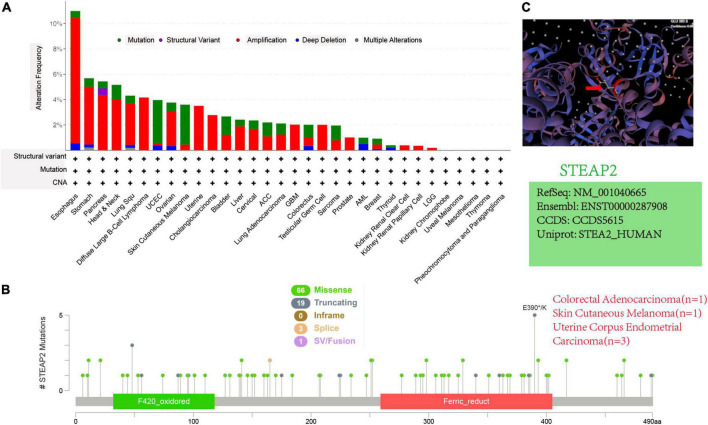
Mutation features of STEAP2 in different tumors of TCGA. **(A,B)** Alteration frequency with mutation type and mutation site. **(C)** Mutation site with the highest alteration frequency (E390) in the 3D structure of STEAP2.

Although structure-based studies help to reveal the function of molecules, no relevant studies have focused on STEAP2 in different tumors. Through cBioPortal, we conducted a preliminary investigation and identified the genetic alteration status of STEAP2 in different tumor samples of TCGA cohorts. As shown in Figure 6A, the “amplification” type was the primary type in most tumors, and the highest alteration frequency of STEAP2 (>10%) occurred in patients with esophageal tumors with “amplification” as the primary type. The “mutation” type of CNA was the primary type in the UCEC and skin cutaneous melanoma cases with an alteration frequency of approximately 4% (Figure 6A). The types, sites, and case numbers of STEAP2 genetic alterations are shown in Figure 6B. Missense mutation of STEAP2 was the main type of genetic alteration, and mutation in E390 of the ferric oxidoreductase domain, which was detected in uterine corpus endometrial carcinoma (*n* = 3), colorectal adenocarcinoma (*n* = 1), and skin cutaneous melanoma (*n* = 1), induced a frameshift mutation of the STEAP2 gene, translation from glutamate (E) to another amino acid at position 390 of the STEAP2 protein, and subsequent STEAP2 protein truncation. The E390 site in the 3D structure of the STEAP2 protein is shown in Figure 6C. The mutation properties of STEAP2 suggest a potential strategy for targeted therapy.

## Discussion

GBM is the most malignant high-grade glioma with poor prognosis and limited therapies (Fisher and Adamson, 2021; Louis et al., 2021). Based on the development of genetics and immunology, the research has focused on the molecular aspects of GBM (Banerjee et al., 2021; Yang et al., 2021; Dai et al., 2022). Treatments targeting crucial biological processes and key proteins have become a new strategy. Copper has multifaceted functions and plays an important role in tumor progression. Abnormal copper homeostasis has been confirmed to impact many processes, such as proliferation, angiogenesis, migration, and pyroptosis (Denoyer et al., 2015; Li, 2020; Shanbhag et al., 2021). However, direct regulation of the intercellular copper concentration is difficult, while targeting copper-related proteins is much easier. Recent research indicated the potential clinical application of copper-related areas. For example, the changed copper metabolism was a new imaging biomarker for metabolic imaging of hepatocellular carcinoma with PET using ^64^CuCl_2_ as a radioactive tracer. Additionally, copper modulation gene therapy based on knockdown of hCtr1 followed by administration of copper chelators has therapeutic potential in cancer (Wachsmann and Peng, 2016). Therefore, increasing studies have focused on copper-related proteins (Banci et al., 2008; Wee et al., 2013; Shanbhag et al., 2019; Tsvetkov et al., 2022), but the functions and detailed mechanisms of these proteins remain unknown.

In recent years, genomics and bioinformatics have made it possible to identify molecular signatures. For example, numerous signatures based on lncRNAs, miRNAs, and mRNAs have been identified for prognostic prediction (Bing et al., 2016; Zhao et al., 2018). We first selected 90 copper-related proteins and determined their subcellular location, and we identified 16 genes with significant differences between tumor and normal samples. Only high expression of STEAP2 improved the OS of GBM patients. Based on these findings, we constructed a prognostic model for all cancer samples and divided all samples into two groups according to risk score. TME is an 18-gene signature that includes genes that reflect an ongoing adaptive Th1 and cytotoxic CD8 T cell response, and it has shown promising results in predicting the response to anti-PD-1/PD-L1 agents. TIS was developed by NanoString Technologies as a clinical-grade assay that provides both quantitative and qualitative information about the TME. The copper-related model, TIDS, and TIS ROC curves revealed that our model was superior to TIDS and TIS. Based on the immunological data from the TCGA cohort, the TMB of 13 differentially expressed copper-related proteins was evaluated.

As mentioned above, high expression of STEAP2 improved the OS of glioma patients. The STEAP family has been confirmed to participate in absorbing and reducing iron and copper as well as to regulate the growth and proliferation of cancer cells. In breast and PRAD, STEAP2 has been reported to suppress cancer progression (Burnell et al., 2018; Yang et al., 2020). Our findings revealed that STEAP2 expression was lower in the high-risk group than in the low-risk group and that the risk factor was inversely associated with STEAP2 expression. Similar to glioma, STEAP2 expression was low in tumor tissues of other cancers and reflected a positive prognosis, which was consistent with previous findings.

It remains unclear whether STEAP2 plays a role in the development of many cancers via common molecular processes. Our literature search did not identify any publications that included a pan-cancer study of STEAP2 from the standpoint of overall tumors. As a result, we evaluated the STEAP2 gene as well as molecular aspects of gene expression and genetic mutation in 33 distinct cancers using data from TCGA and CPTAC databases. However, there was no entry describing the STEAP2 structural mutation. We utilized cBioportal and found that the “amplification” form of STEAP2 mutation was the most common in the majority of cancers, indicating a novel mutation responsible for STEAP2 function loss, which should be investigated in the future.

Based on the predictive values of the copper-related proteins, we developed a prognostic model. Further validation of our model revealed associations of the risk score with clinical prognosis, immune cell infiltration, and TMB. The expression of STEAP2 in tumor tissues was lower than that in normal tissues, indicating a better prognosis. A pan-cancer study has predicted E390 to be a crucial mutation hotspot due to its function in the prognosis of various tumors.

The present study had various advantages. First, the signature was thoroughly validated and assessed across numerous datasets, demonstrating its resilience and reliability. Second, an extensive and in-depth study was conducted on various topics, including the relationship between risk score and immune cells as well as TMB. Third, data from various databases were analyzed, laying the groundwork for future studies. Nonetheless, the present study had several limitations. The function of STEAP2 in immune infiltration was not thoroughly explored, and STEAP2 staining of clinical samples could further demonstrate the downregulation of STEAP2 expression in glioma.

Finally, our findings lay the groundwork for future research focusing on copper-related proteins and their immunological milieu to improve prognosis and immunotherapy responses.

## Data availability statement

The original contributions presented in this study are included in the article/supplementary material, further inquiries can be directed to the corresponding authors.

## Ethics statement

Ethical review and approval was not required for the study on human participants in accordance with the local legislation and institutional requirements. Written informed consent for participation was not required for this study in accordance with the national legislation and the institutional requirements.

## Author contributions

XW, MH, and SC designed the project and performed the analysis. XW, MH, SC, and YS wrote the manuscript. MH and XW provided formal analysis support. BH and RT supervised the project. All authors contributed to the article and approved the submitted version.

## Footnotes

1
https://tcga-data.nci.nih.gov/tcga

2
http://caintegrator-info.nci.nih.gov/rembrandt

3
http://geneontology.org

4
http://www.genome.jp/kegg/

5
https://cibersort.stanford.edu/

6
http://timer.cistrome.org/

7
http://ualcan.path.uab.edu/index.html

8
https://www.cbioportal.org/

9
https://swissmodel.expasy.org/

10
https://www.uniprot.org/

11
http://www.cgga.org.cn/analyse/RNA-data.jsp
